# Factors influencing person-centered care among nurses in long-term care hospitals in South Korea: a cross-sectional study

**DOI:** 10.7586/jkbns.25.044

**Published:** 2025-08-22

**Authors:** Suhye Kwon, Hyochol Brian Ahn, Miseon Bang

**Affiliations:** 1 College of Nursing, Kosin University, Busan, Korea; 2 College of Nursing, University of Arizona, Tucson, USA; 3 Department of Nursing, Kyungnam College of Information and Technology, Busan, Korea

**Keywords:** Aged, Long-term care, Nurses, Patient-centered care

## Abstract

**Purpose:**

This study examined levels of compassion competence, professional self-concept, and nursing organizational culture among nurses in long-term care hospitals (LTCHs) and analyzed their influence on person-centered care (PCC) in South Korea. The aim was to provide foundational data to support strategies for improving PCC.

**Methods:**

A cross-sectional descriptive survey was conducted with 201 nurses from 10 LTCHs in Busan, South Korea. Validated instruments were used to assess compassion competence, professional self-concept, nursing organizational culture, and PCC. Data were analyzed using descriptive statistics, the t-test, analysis of variance, Pearson correlation coefficients, and hierarchical multiple regression using SPSS version 25.0.

**Results:**

The mean PCC score was 3.44 out of 5, indicating a moderate level. The levels of compassion competence (M = 3.87 out of 5) and professional self-concept (M = 5.97 out of 8) were also moderate. PCC was positively correlated with compassion competence, professional self-concept, and specific subdomains of organizational culture. Hierarchical regression identified compassion competence (β = 0.43, *p* < .001), innovation-oriented culture (β = 0.19, *p* = .006), and satisfaction with work (β = 0.13, *p* = .042) as significant predictors of PCC, accounting for 48.0% of the variance.

**Conclusion:**

Compassion competence, innovation-oriented nursing organizational culture, and satisfaction with work significantly influenced PCC among nurses in LTCHs. Healthcare institutions should actively implement strategies to enhance nurses’ compassion competence and promote a supportive, innovative workplace culture and environment to improve PCC.

## INTRODUCTION

With the advancement of medical technology and the extension of average life expectancy, the proportion of order populations is rapidly increasing worldwide. In South Korea, the proportion of the population aged 65 and older reached 19.4% in 2024, officially categorizing the country a super-aged society [1]. This proportion is expected to rise to 25.5% by 2030, 34.4% by 2040, and exceed 44% by 2060 [1]. This demographic shift has contributed to an increase in chronic degenerative diseases among older adults, thereby intensifying the demand for long-term care services [2]. Long-term care hospitals (LTCHs), established in 1994 to support the aging population, increased to 1,437 by 2022 following the launch of the Long-Term Care Insurance System in 2008, and provide holistic, integrated care led by skilled nurses [3]. However, the rapid proliferation of these facilities has raised serious concerns about the quality of care, especially for older adults facing physical and mental challenges. Therefore, improving the long-term care environment has become an urgent priority [4].

Creating an environment that enables person-centered care (PCC) for older adults residing in LTCHs is essential [5]. PCC refers to ethical nursing practice that respects the values, autonomy, and choices of individuals, and emphasizes collaborative decision-making between patients and healthcare providers [6]. This approach not only enhances the quality of care for elderly patients but also reduces feelings of helplessness, improves patient satisfaction, and contributes to better treatment outcomes [7]. It also provides caregivers with the opportunity to deliver individualized care and strengthen job satisfaction through effective communication with patients, ultimately contributing to improved quality of nursing services [6]. Thus, PCC is a critical concept in geriatric nursing within LTCHs, requiring in-depth exploration and development of strategies to enhance nurses’ competencies in delivering person-centered care.

One of the key elements in practicing PCC is the compassion competence of nurses. Compassion can be understood as an emotional response characterized by sensitivity to another’s suffering and the desire to alleviate that suffering [8]. It goes beyond mere emotional resonance, requiring an active stance to understand a person’s situation from their perspective. Compassion competence encompasses the knowledge, skills, and attitudes that enable nurses to holistically understand their patients and is considered a critical component of overall nursing competence [9]. Nurses with high compassion competence are able to detect subtle changes in a patient’s condition, respond promptly to their needs, and communicate effectively with family members and interdisciplinary teams to provide integrated care [10]. Such competence allows nurses to build therapeutic relationships with patients and tailor care to the individual's circumstances [11]. Moreover, a deep understanding of care recipients enhances clinical performance in terms of precision and efficiency, which in turn contributes positively to the overall quality of nursing services [12,13]. Therefore, compassion competence among long-term care hospital nurses is expected to be closely linked to person-centered care, and systematic investigation of this relationship holds significant academic and practical value.

Another crucial factor is the nurse’s professional self-concept, which reflects their perception and beliefs about themselves as nursing professionals [14]. A strong professional self-concept contributes to problem-solving capabilities, confidence, and effective communication, while a weak self-concept may lead to emotional fatigue and hinder compassion interactions [14,15]. Prior studies have shown that professional self-concept is positively associated with geriatric care performance [16], suggesting a potential connection with PCC. However, there is a lack of research that specifically examines this relationship, highlighting the need for further inquiry.

Nursing organizational culture refers to the shared beliefs and values held by members of a nursing organization and is a major factor influencing individual behavior within the organization [17]. In particular, organizational culture possesses the potential to either facilitate or constrain the implementation of PCC, suggesting that the practice level of such care may vary depending on the specific cultural characteristics present [18,19]. Therefore, identifying the types of organizational culture that support or hinder PCC is critically important. LTCHs, which have been established in greater numbers compared to other healthcare facilities, are expected to exhibit diverse organizational cultures due to their varying institutional characteristics [18]. However, research on the nursing organizational culture within LTCHs remains limited. Likewise, studies investigating the relationship between nursing organizational culture and PCC in these settings are also scarce. As such, exploring the types of nursing organizational culture that can effectively support the delivery of PCC to older adults residing in LTCHs holds significant value for shaping and improving environments conducive to person-centered nursing practice.

Previous studies on PCC have mainly focused on nurses in general or acute care settings, such as small hospitals [20] and tertiary medical centers [21]. While some research has been conducted among LTCH nurses [22–24], it has primarily examined moral distress, empowerment, work environments, spiritual well-being, and geriatric care stress as influencing factors. Comprehensive investigations that analyze the roles of compassion competence, professional self-concept, and organizational culture in relation to PCC are still limited. Therefore, this study aims to assess the levels of compassion competence, professional self-concept, nursing organizational culture, and PCC among LTCH nurses, examine the interrelationships between these variables, and identify key factors influencing PCC, ultimately providing foundational data to develop effective strategies for enhancing PCC practice.

## METHODS

### 1. Study design

This study employed a cross-sectional descriptive design to investigate the levels of compassion competence, professional self-concept, nursing organizational culture, and PCC among long-term care hospital nurses. Additionally, the study aimed to explore the correlations among these variables and identify factors influencing person-centered care.

### 2. Participants

Participants were 201 nurses working at ten long-term care hospitals in Busan metropolitan city who voluntarily agreed to participate after being informed of the study’s purpose. The minimum required sample size was determined using G*Power 3.1.9 [25], with a significance level of .05, power of .95, and medium effect size of .15 based on previous research [24], resulting in a required sample of at least 184 for multiple regression analysis with 12 predictors. A total of 201 responses were collected within the designated period. All participants completed the survey in full, and no cases were excluded; thus, all responses were used in the final analysis, resulting in a dropout rate of 0%.

### 3. Instruments

General characteristics of the participants were assessed with 13 items, including age, gender, marital status, religious affiliation, educational level, perceived health status, clinical experience, job position, work pattern, number of hospital beds, number of patients per duty, satisfaction with work, and satisfaction with salary. The survey also included 13 items on PCC, 17 items on compassion competence, 14 items on professional self-concept, and 20 items on nursing organizational culture, 77 items in total.

#### 1) PCC

PCC was assessed using the Korean version of the Person-Centered Care Assessment Tool, originally developed by Edvardsson et al. [26] and validated by Tak et al. [27]. It consists of 13 items across two subscales: individualized care (seven items) and organizational and environmental support (six items). Each item is rated on a 5-point Likert scale (1=strongly disagree to 5=strongly agree), with higher scores indicating a higher level of PCC. Cronbach’s α was .86 in the prior study [27] and .84 in this study.

#### 2) Compassion competence

Compassion competence was measured using the Compassion Competence Scale developed by Lee [9], comprising 17 items across three subdomains: communication (eight items), sensitivity (five items), and insight (four items). Responses are rated on a 5-point Likert scale (1=strongly disagree to 5=strongly agree), with higher scores reflecting greater compassion competence. Cronbach’s α was .91 in the original study [9] and .90 in the present study.

#### 3) Professional self-concept

Professional self-concept was assessed using the short version of the Nurse’s Self-Concept Instrument developed by Angel [28] and validated by Ryu [29]. The tool includes 14 items in four domains: knowledge (four items), caring (three items), relationships with colleagues (three items), and leadership (four items). Each item is rated on an 8-point Likert scale (1 = strongly disagree to 8 = strongly agree), with higher scores indicating a stronger professional self-concept. Cronbach’s α was .94 in the previous study [29] and .95 in this study.

#### 4) Nursing organizational culture

Evaluated using a tool developed by Kim [30], consisting of 20 items in four subdomains: relation-oriented (five items), innovation-oriented (six items), hierarchy-oriented (five items), and task-oriented culture (four items). Items are rated on a 5-point Likert scale (1=strongly disagree to 5=strongly agree), and higher scores indicate greater recognition of the corresponding cultural attributes. Cronbach’s α was .88 in the original study [30] and .82 in this study.

### 4. Data collection

Data were collected from December 6 to 30, 2022. The researcher obtained prior approval and cooperation from the nursing department administrators at the participating LTCHs. Recruitment notices containing the survey Uniform Resource Locator or Quick Response code were posted on the institutions’ online communities and smartphone messenger applications, directing participants to the survey’s initial access screen. Nurses who voluntarily agreed to participate accessed the survey via the provided link and responded only once within the designated period. A total of 201 completed questionnaires were collected during the data collection period and used for the final analysis.

### 5. Data analysis

The collected data were analyzed using SPSS version 25.0 software (IBM Corp., Armonk, NY, USA), and the specific procedures were as follows: participants’ general characteristics were analyzed using frequencies, percentages, means, and standard deviations. The levels of compassion competence, professional self-concept, nursing organizational culture, and PCC were analyzed using means and standard deviations. Differences in PCC according to general characteristics were examined using independent t-tests and one-way analysis of variance, with Scheffé tests conducted for post-hoc comparisons. Pearson correlation coefficients were used to analyze relationships among compassion competence, professional self-concept, nursing organizational culture, and PCC. Factors influencing PCC were identified using hierarchical multiple regression analysis.

### 6. Ethical considerations

Prior to commencing the study, approval was obtained from the Institutional Review Board (IRB) of Kosin University (IRB No. 2022-0062) to ensure the protection of participants’ rights and personal information. To uphold ethical standards, the researcher provided participants with an information sheet and informed consent form detailing the purpose, procedures, potential risks and benefits, compensation, data privacy and protection, the right to withdraw, and access by relevant institutions. For online data collection, participants were shown the study description and consent form before beginning the survey, and only those who clicked the “Agree” button were able to proceed. After completing the survey, all participants who provided their mobile phone numbers for compensation purposes received mobile gift vouchers, and the collected contact information was immediately deleted.

## RESULTS

### 1. General characteristics of participants

Participants had a mean age of 43.79 ± 9.95 years. The largest age group was those in their 40s (n = 67, 33.3%), followed by those in their 50s (n = 60, 29.9%). Most were woman (n = 189, 94.0%), married (n = 138, 68.7%), and had no religious affiliation (n = 111, 55.2%). A bachelor’s degree was the highest level of education for 47.3% (n = 95). “neutral” was the most common perceived health status (n = 97, 48.3%). Total clinical experience averaged 15.84 ± 9.64 years, with the largest group having over 25 years (n = 47, 23.4%). Most were staff nurses (n = 162, 80.6%) and worked fixed shifts (n = 119, 59.2%). Regarding hospital size, 42.8% (n = 86) were at facilities with over 300 beds. The most common patient load was over 60 per shift (n = 81, 40.3%). In terms of satisfaction, most were satisfied with their job (n = 89, 44.3%) but dissatisfied with salary (n = 93, 46.3%) (Table 1).

### 2. Levels of compassion competence, professional self-concept, nursing organizational culture, and PCC

Participants’ compassion competence scored an average of 3.87 ± 0.44 on a 5-point scale. The average score for professional self-concept was 5.97 ± 0.99 on an 8-point scale. Regarding subscales of nursing organizational culture, the average scores were as follows: relation-oriented culture, 3.53 ± 0.69; hierarchy-oriented culture, 3.53 ± 0.53; innovation-oriented culture, 3.10 ± 0.71; and task-oriented culture, 2.95 ± 0.65—all measured on a 5-point scale. The level of PCC was also assessed using a 5-point scale and showed a mean score of 3.44 ± 0.56 (Table 2).

### 3. Differences in PCC by general characteristics

There were significant differences in levels of PCC according to perceived health status (F = 7.01, *p* = .003), satisfaction with work (F = 22.77, *p* < .001) and satisfaction with salary (F = 4.00, *p* = .020). Post hoc analysis revealed that participants who perceived their health as “good” reported higher levels of PCC compared to those who responded with “neutral” or “poor.” Likewise, those who reported being “satisfied” with their job and salary demonstrated higher levels of PCC than those who responded “neutral” or “dissatisfied” (Table 1).

### 4. Correlations among variables

An analysis of the correlations among participants’ study variables revealed that PCC was positively and significantly correlated with compassion competence (r = .62, *p* < .001), professional self-concept (r = .53, *p* < .001), and two subdimensions of nursing organizational culture—innovation-oriented culture (r = .48, *p* < .001) and relation-oriented culture (r = .54, *p* < .001) (Table 3).

### 5. Factors influencing PCC

To identify the factors influencing PCC, a hierarchical multiple regression analysis was conducted. The independent variables included four factors that showed significant correlations with PCC— compassion competence, professional self-concept, and two subdimensions of nursing organizational culture (innovation-oriented and relation-oriented cultures)—as well as three general characteristics that demonstrated significant group differences in PCC: perceived health status, job satisfaction, and salary satisfaction. Multicollinearity among the independent variables was assessed, and no issues were detected. The tolerance values ranged from .44 to .86 (all > 0.1), and the variance inflation factors ranged from 1.19 to 2.29 (all < 10). Residual analysis showed a Durbin-Watson statistic of 1.84, indicating no autocorrelation in residuals.

In Step 1 of the hierarchical regression, the dummy-coded general characteristics—perceived health status, job satisfaction, and salary satisfaction—were entered. The model was statistically significant (F = 15.31, *p* < .001), with job satisfaction (β = 0.37, *p* < .001) emerging as a significant predictor, explaining 18% of the variance in PCC.

In Step 2, after controlling for these general characteristics, the four study variables were entered. The final model was statistically significant (F = 27.81, *p* < .001), and three variables were identified as significant predictors of PCC: compassion competence (β = 0.43, *p* < .001), innovation-oriented culture (β = 0.19, *p* = .006), and satisfaction with work (β = 0.13, *p* = .042). Together, these variables accounted for 48.0% of the variance in PCC (Table 4).

## DISCUSSION

This study aimed to examine the levels of compassion competence, professional self-concept, nursing organizational culture, and PCC among long-term care hospital nurses, and to identify the factors influencing PCC.

The average score for PCC among participants was 3.44 out of 5, similar to scores reported in previous studies using the same instrument (3.42) [31] and others (3.44) [32], indicating a moderate level of PCC among nurses in LTCHs. This score is slightly lower than those reported in Western countries such as Sweden (3.75) [32], which may be due to cultural differences, institutional support levels, and nursing workforce structures. Comparative studies across countries are needed to explore these differences systematically. With East Asian countries entering aged societies and the rapid aging trend continuing, LTCHs’ expansion has raised concerns about nursing care quality [33]. Despite growing global interest in PCC and quality management in long-term care settings, research on the conceptualization and management systems of PCC within the Korean cultural context remains insufficient [31]. Nurses in LTCHs caring for functionally impaired older adults have opportunities to provide individualized care through PCC and enhance job satisfaction via effective communication, contributing to improved nursing quality [6]. Therefore, strategies such as continuing education and nursing management improvements are needed to strengthen PCC capacity and practice.

The average compassion competence score among nurses in LTCHs was 3.87 out of 5, indicating a slightly above-average level. Compared to previous findings, this score was somewhat lower than that of care workers (3.95 out of 5) [34], and marginally higher than that of general hospital nurses (3.83) [21], though the difference may not be practically significant.

These results may reflect the unique characteristics of long-term care nursing. While care workers may have more frequent opportunities for compassionate interaction due to their direct caregiving roles, nurses in LTCHs —unlike those in acute care settings—often have more time to engage with patients, allowing for deeper understanding and compassionate connection. Compassion competence enhances clinical performance by improving understanding of patients’ needs, thereby contributing to nursing quality [12,13]. Therefore, efforts to enhance compassion competence among long-term care nurses are essential and should be prioritized in both educational and organizational strategies.

The professional self-concept score was 5.97 out of 8, similar to a previous study using the same instrument (6.02) [29], indicating an above-midpoint level. Compared to nurses in small- to medium-sized hospitals (5.81) [20], the score was slightly higher, possibly due to participants’ older age and longer clinical experience [16]. LTCH nurses often collaborate with other professionals and supervise nursing assistants, enhancing leadership roles and decision-making, which may strengthen professional self-concept. Prior research shows professional self-concept positively influences geriatric nursing performance and quality [16], and this study also found a significant correlation with PCC. Therefore, multifaceted strategies such as expanding autonomy, leadership opportunities, and interdisciplinary collaboration are essential to maintain and enhance professional self-concept among LTCH nurses [29].

In this study, participants perceived relation-oriented culture as the most prominent type of nursing organizational culture, with an average score of 3.59 out of 5, followed by hierarchy-oriented, innovation-oriented, and task-oriented cultures. This finding aligns with a recent study that used the same instrument to assess organizational culture among long-term care hospital nurses, in which relation-oriented culture also ranked highest at 3.69, with the same order observed among the subdomains [18]. These results suggest that nurses in LTCHs most strongly identify with a relation-oriented organizational culture. In contrast, previous studies involving nurses in tertiary and university hospitals have reported hierarchy-oriented culture as the most dominant [35]. This discrepancy may be attributed to the nature and scale of acute care hospitals, where urgent, life-threatening situations are more common and rapid, top-down decision-making is often required—fostering a stronger hierarchy-oriented culture. On the other hand, LTCHs, which tend to be smaller in scale, may emphasize flexibility and interpersonal relationships, making relation-oriented culture more salient. Interestingly, in this study, hierarchy-oriented culture was also perceived relatively strongly. This may be due to the demographic characteristics of the participants, many of whom were middle-aged (over 50 years old) with an average of 16 years of clinical experience. Such individuals may have already adapted to structured systems and place greater value on rules and protocols. Given that perceptions of organizational culture can vary depending on the size and characteristics of the institution, further research is needed to better understand the organizational culture types specific to long-term care hospital nurses, an area that remains underexplored.

Analysis of factors associated with differences in the level of PCC revealed that perceived health status, job satisfaction, and salary satisfaction were significant. In other words, nurses with better perceived health and higher satisfaction with their work and pay demonstrated higher levels of PCC. This finding offers important implications, as it suggests that overall quality of life and occupational satisfaction directly influence the quality of nursing practice. These results align with previous research indicating that physical health and psychological well-being contribute to greater happiness among nurses [19]. Particularly, nurses who perceive their own health and work environment positively are more likely to provide responsible and authentic care, which is a core component of person-centered nursing. Previous Korean [36] and international [37] studies similarly report positive associations between nursing work satisfaction and PCC-supportive environments. This suggests PCC is influenced by complex organizational and systemic contexts beyond individual characteristics. Thus, strategies such as wellness programs, clear job roles, and improved compensation are needed to promote PCC practice among LTCH nurses [37].

Hierarchical multiple regression analysis conducted in this study identified compassion competence, innovation-oriented culture and job satisfaction as significant predictors of PCC among nurses in LTCHs. The most influential factor was compassion competence, consistent with previous research involving long-term care nurses [31], which also found it to be the strongest predictor. Additional studies have reported that nurses in these settings experience a wide range of emotions during interactions with patients, and that their compassion competence is significantly correlated with the quality of geriatric nursing practice [38]. This suggests that compassion competence plays a critical role in enhancing understanding and emotional support for patients, thereby strengthening the implementation of PCC. Given that patients in LTCHs often suffer from chronic illnesses, degenerative conditions, or cognitive impairments, emotional support and compassion are essential components of care—beyond routine medical interventions. Compassion-enhancement programs have proven effective in improving compassion competence [39], underscoring the need for structured educational programs to promote PCC.

The second factor found to influence PCC in this study was innovation-oriented culture, a subdomain of nursing organizational culture. This finding aligns with previous research involving nurses in LTCHs [18] and is consistent with studies on long-term care facility workers [19,34], which also identified innovation-oriented culture as a key factor affecting PCC [40]. Interestingly, while innovation-oriented culture did not significantly influence PCC among nurses in tertiary hospitals [40], it consistently emerged as a significant factor in studies involving long-term care nurses and care workers [18,34]. This discrepancy may be attributed to differences in organizational environments and job characteristics. In particular, innovation-oriented culture in long-term care settings may foster a shift away from standardized, procedural care toward more individualized approaches that respect patient preferences and autonomy—thereby cultivating an environment conducive to PCC. Recently, the nursing paradigm has been shifting from provider-centered, disease-focused care to PCC that reflects the needs and preferences of long-term care recipients [40,41]. Given that PCC emphasizes tailored nursing services based on patients’ choices, values, and needs, fostering a creative and flexible care environment through innovation-oriented culture is essential. Moreover, without adequate awareness and support from leadership, nurses may face challenges in implementing PCC effectively [41]. Therefore, nurse managers and leaders in LTCHs should actively promote innovation-oriented culture to establish a strong foundation for PCC practice.

The third influencing factor was job satisfaction. Although prior studies on long-term care nurses haven’t directly identified this, previous research on nursing environments and job stress [42] supports that positive work perceptions may foster PCC. This aligns with studies linking supportive care environments and higher job satisfaction in long-term care settings [36], including international cases like the Netherlands [37]. These findings highlight that PCC is shaped not only by individual traits but also by institutional and systemic conditions. Accordingly, improving work conditions, roles, and benefits is essential to enhance job satisfaction and promote PCC. Meanwhile, professional self-concept did not significantly affect PCC. Given limited research in this area, future comparative studies would be meaningful.

Based on the findings, nurses in LTCHs showed higher engagement in PCC when they had greater compassion competence, perceived an innovation-oriented organizational culture, and were more satisfied with their jobs. While earlier studies explored PCC in long-term care, this study highlights practical factors—such as perceived health, job, and salary satisfaction—alongside compassion competence and organizational culture. These results emphasize the importance of both individual development and organizational support to enhance PCC in LTCHs.

## CONCLUSION

This study aimed to provide foundational data for developing interventions to enhance PCC among nurses in LTCHs by analyzing the effects of compassion competence, professional self-concept, and nursing organizational culture. The findings revealed that compassion competence, innovation-oriented nursing organizational culture, and job satisfaction were significant factors influencing the practice of PCC. To enhance PCC among nurses in LTCHs, both short- and long-term strategies are needed to strengthen compassion competence. In addition, fostering an innovative nursing organizational culture is essential. Systematic measures must also be developed to improve job satisfaction among these nurses.

This study has several limitations. First, participants were selected through convenience sampling from LTCHs in a single region, which limits the generalizability of the findings to nurses nationwide. Second, as data were collected using a structured questionnaire on PCC, the study may not have fully captured the range of actual care activities performed by nurses in practice. Future research should include diverse countries, regions, and healthcare institutions to conduct more in-depth and comprehensive analyses of the factors influencing PCC. Additionally, it is recommended that future studies develop and implement compassion-enhancement programs tailored to the context of LTCHs and evaluate their effectiveness.

## Figures and Tables

**Table 1.** Differences in Person-centered Care among Long-term Care Hospital Nurses According to General Characteristics (N = 201)

Characteristics	Categories	n (%)/M ± SD	Person-centered care	
			M ± SD	t/F (*p*)/Scheffé
Age (year)	< 30	21 (10.4)	3.41 ± 0.60	1.46 (.216)
	30~39	46 (22.9)	3.44 ± 0.61	
	40~49	67 (33.3)	3.38 ± 0.53	
	50~59	60 (29.9)	3.46 ± 0.52	
	≥ 60	7 (3.5)	3.90 ± 0.39	
		43.79 ± 9.95		
Sex	Women	189 (94.0)	3.42 ± 0.56	−0.90 (.369)
	Men	12 (6.0)	3.57 ± 0.45	
Marital status	Single	63 (31.3)	3.34 ± 0.58	1.59 (.115)
	Married	138 (68.7)	3.47 ± 0.54	
Religion	Yes	90 (44.8)	3.44 ± 0.51	0.21 (.837)
	No	111 (55.2)	3.42 ± 0.59	
Education	Associate’s degree	84 (41.8)	3.50 ± 0.54	1.15 (.320)
	Bachelor's degree	95 (47.3)	3.38 ± 0.54	
	≥ Master's degree	22 (10.9)	3.43 ± 0.67	
Perceived health status	Poor^a^	26 (12.9)	3.21 ± 0.62	7.01 (.003)
	Neutral^b^	97 (48.3)	3.36 ± 0.50	a,b < c
	Good^c^	78 (38.8)	3.61 ± 0.56	
Length of work as a nurse (year)	< 5	33 (16.4)	3.54 ± 0.50	2.09 (.069)
	5~9	35 (17.4)	3.22 ± 0.49	
	10~14	22 (11.0)	3.29 ± 0.69	
	15~19	28 (13.9)	3.41 ± 0.47	
	20~24	36 (17.9)	3.51 ± 0.59	
	≥ 25	47 (23.4)	3.55 ± 0.54	
		15.84 ± 9.64		
Position	Staff	162 (80.6)	3.39 ± 0.54	−1.95 (.052)
	Manager	39 (19.4)	3.59 ± 0.59	
Type of duty	Shift duty	82 (40.8)	3.36 ± 0.53	1.49 (.138)
	Fixed duty	119 (59.2)	3.48 ± 0.57	
Number of beds in hospital	< 100	10 (5.0)	3.49 ± 0.46	1.11 (.348)
	100~199	41 (20.4)	3.49 ± 0.55	
	200~299	64 (31.8)	3.33 ± 0.53	
	≥ 300	86 (42.8)	3.48 ± 0.59	
Number of assigned patients per shift	< 20	14 (7.0)	3.70 ± 0.52	1.33 (.266)
	20~39	39 (19.4)	3.41 ± 0.65	
	40~59	67 (33.3)	3.38 ± 0.53	
	≥ 60	81 (40.3)	3.44 ± 0.53	
		49.30 ± 18.90		
Satisfaction with work	Dissatisfied^a^	26 (12.9)	3.09 ± 0.51	22.77 (< .001)
	Neutral^b^	86 (42.8)	3.27 ± 0.49	a,b < c
	Satisfied^c^	89 (44.3)	3.70 ± 0.51	
Satisfaction with salary	Dissatisfied^a^	93 (46.3)	3.36 ± 0.58	4.00 (.020)
	Neutral^b^	75 (37.3)	3.42 ± 0.49	a < c
	Satisfied^c^	33 (16.4)	3.68 ± 0.61	

M = Mean; SD = Standard deviation.

**Table 2.** Levels of Compassion Competence, Professional Self-concept, Nursing Organizational Culture, and Person-centered Care among Participants (N = 201)

Variables	M ± SD	Min	Max	Range
Compassion competence	3.87 ± 0.44	2.35	5.00	1∼5
Professional self-concept	5.97 ± 0.99	3.00	8.00	1∼8
Nursing organizational culture				1∼5
Innovation-oriented culture	3.10 ± 0.71	1.00	5.00	
Relation-oriented culture	3.53 ± 0.69	1.20	5.00	
Hierarchy-oriented culture	3.53 ± 0.53	1.60	5.00	
Task-oriented culture	2.95 ± 0.65	1.00	4.75	
Person-centered care	3.44 ± 0.56	2.08	4.85	1∼5

M = Mean; SD = Standard deviation; Min = Minimum; Max = Maximum.

**Table 3.** Correlations among Compassion Competence, Professional Self-concept, Nursing Organizational Culture, and Person-centered Care (N = 201)

Variables		1	2	3-1	3-2	3-3	3-4	4
		r (*p*)						
1. Compassion competence		1	-	-	-	-	-	-
2. Professional self-concept		.69 (< .001)	1	-	-	-	-	-
3. Nursing organizational culture	3-1. Innovation-oriented culture	.34 (.032)	.44 (< .001)	1	-	-	-	-
	3-2. Relation-oriented culture	.49 (.001)	.48 (< .001)	.63 (< .001)	1	-	-	-
	3-3. Hierarchy-oriented culture	.04 (.619)	−.02 (.801)	−.17 (.016)	.05 (.505)	1	-	-
	3-4. Task-oriented culture	.08 (.238)	.23 (< .001)	.46 (< .001)	.20 (.004)	.19 (.007)	1	-
4. Person-centered care		.62 (< .001)	.53 (< .001)	.48 (< .001)	.54 (< .001)	−.05 (.453)	.07 (.331)	1

**Table 4.** Factors Influencing Person-centered Care (N = 201)

Variables		Model 1					Model 2				
		B	SE	*β*	t	*p*	B	SE	*β*	t	*p*
Perceived health status		0.11	0.08	0.09	1.32	.187	0.03	0.06	0.03	0.43	.667
Satisfaction with work		0.42	0.08	0.37	5.29	< .001	0.14	0.07	0.13	2.05	.042
Satisfaction with salary		0.07	0.1	0.05	0.71	.477	0.07	0.08	0.02	0.31	.754
Compassion competence							0.55	0.10	0.43	5.83	< .001
Professional self-concept							0.01	0.04	0.02	0.21	.835
Nursing organizational culture	Innovation-oriented culture						0.03	0.01	0.19	2.78	.006
	Relation-oriented culture						0.02	0.01	0.14	1.95	.052
Adjusted R^2^		.18					.48				
R^2^ change							.30				
F (*p*)		15.31 (< .001)					27.81 (< .001)				
Durbin-Watson		1.84									

Perceived health status (0 = poor, neutral, 1 = good); Satisfaction with work (0 = dissatisfied, neutral, 1 = satisfied); Satisfaction with salary (0 = dissatisfied, neutral, 1 = satisfied). SE = Standard error.

## Data Availability

The dataset supporting the conclusions is available from the corresponding author on reasonable request.
